# Acute effects of motor learning models on technical efficiency in strength-coordination exercises: a comparative analysis of Olympic snatch biomechanics in beginners

**DOI:** 10.5114/biolsport.2025.141662

**Published:** 2024-07-31

**Authors:** Achraf Ammar, Atef Salem, Marvin Leonard Simak, Fabian Horst, Wolfgang I. Schöllhorn

**Affiliations:** 1 Department of Training and Movement Science, Institute of Sport Science, Johannes Gutenberg-University Mainz, Mainz, Germany; 2 Research Laboratory, Molecular Bases of Human Pathology, LR19ES13, Faculty of Medicine of Sfax,University of Sfax, Sfax 3029, Tunisia; 3 High Institute of Sport and Physical Education of Sfax, University of Sfax, Sfax, Tunisia

**Keywords:** Motor learning, Kinematics, Kinetics, Whole-body movement, Weightlifting

## Abstract

Despite the development of various motor learning models over many decades, the question of which model is most effective under which conditions to optimize the acquisition of skills remains a heated and recurring debate. This is particularly important in connection with learning sports movements with a high strength component. This study aims to examine the acute effects of various motor learning models on technical efficiency and force production during the Olympic snatch movement. In a within-subject design, sixteen highly active male participants (mean age: 23.13 ± 2.09 years), who were absolute beginners regarding the learning task, engaged in randomized snatch learning bouts, consisting of 36 trials across different learning models: differential learning (DL), contextual interference (serial, sCI; and blocked, bCI), and repetitive learning (RL). Kinematic and kinetic data were collected from three snatch trials executed following each learning bout. Discrete data from the most commonly monitored biomechanical parameters in Olympic weightlifting were analyzed using inferential statistics to identify differences between learning models. The statistical analysis revealed no significant differences between the learning models across all tested parameters, with p-values ranging from 0.236 to 0.99. However, it was observed that only the bouts with an exercise sequence following the DL model resulted in an average antero-posterior displacement of the barbell that matched the optimal displacement. This was characterized by a mean positive displacement towards the lifter during the pulling phases, a negative displacement away from the lifter in the turnover phase, and a return to positive displacement in the catch phase. These findings indicate the limited acute impact of the exercise sequences based on the three motor learning models on Olympic snatch technical efficiency in beginners, yet they hint at a possible slight advantage for the DL model. Coaches might therefore consider incorporating the DL model to potentially enhance technical efficiency, especially during the early stages of skill acquisition. Future research, involving even bigger amounts of exercise noise, longer learning periods, or a greater number of total learning trials and sessions, is essential to verify the potential advantages of the DL model for weightlifting technical efficiency.

## INTRODUCTION

Since the inception of motor learning and sports pedagogy, the focus has been more on understanding what, why, and how much variation contributes to optimal enhancement for individual learners, than on questioning whether such enhancement occurs. Historically, the repetitive learning (RL) model, traced back to Plato [1] emphasized the role of copying a role model and repeating correct trials, aiming to enable the learner to perform certain skills with greater precision and accuracy. The underlying belief is that by repeatedly practising accurate movements, the motor system becomes more efficient [2]. Following the theory of behaviourism, efforts were primarily directed towards inducing changes in movement behaviour by manipulating external stimuli through external variations (e.g., instructions, prescriptions, material aid, etc.) [2]. With the advent of the cognitive turn [3] in psychology coupled with the emergence of cybernetics [4] in the second half of the 20^th^ century, a shift of variations was observable from the system’s surroundings towards the system itself “the learner”, although similar developments were observable in sports pedagogy within reform pedagogy earlier [5, 6]. This shift involved the differentiation of tasks through models such as the Variability of Practice (VP) model, where the invariants (e.g., relative timing, relative forces, etc.) are held constant while varying the variable parameters (e.g., absolute timing, absolute forces, etc.) [7, 8], and contextual interference (CI) learning, where the schedule is varied, encompassing the temporal structuring of the learning process [9, 10].

In these models, variation of boundary conditions involved the manipulation of the person (e.g., inducing time pressure to place stress on them [11]), varying the tasks (e.g., employing the same generalized motor program with varying variable parameters), and manipulating the contextual and/or environmental aspects (e.g., using different weights for throwing bean bags over varying distances [8, 9, 11]. All these manipulations were meticulously examined concerning their impact on the learner, as it was only the learner who had to and could adapt. Nevertheless, the entire learning process was embedded in an external feedback loop, wherein the learning process was externally controlled through augmented information on correctness or errors and movement restrictions through material aids. Especially within the domain of sports, there was often a standard role model of correct performance that always applied to all learners, with individual variations only tolerated at the highest level of performance, formalized by the principle of individualization [12]. In this context, it is important to differentiate this from the earlier principle of individuality [13, 14]. In contrast to the orientation on a previously set value (reference movement), the stochastic resonance model as suggested within the differential learning (DL) model [14] is derived from modern physics and occurs adaptively within a time-delayed feedback loop when a system is exposed to an external force or oscillation that adjusts to its time-shifted inherent natural frequency [15]. Considering the diverse range of experiences and ever-shifting boundary conditions affecting learners, such as emotions, fatigue, chronobiology, and aging the resonance principle necessitates an appropriate adjustment of external forces to achieve situated resonance. This concept has recently been termed adaptive stochastic resonance [16]. However, it was originally employed by the DL model, which first emerged publicly in the late 1990s [17, 18]. Indeed, the DL model argues that effective learning necessitates a more generalized introduction of differences achieved through the integration of stochastic perturbations or noise [19, 20] that should be tuned to the learner’s individual and situational characteristics throughout the learning process [2, 21, 22].

Despite the development of these various learning models (RL, VP, CI, and DL) over many decades and even though only the RL and DL models have been originally designed for the sports field [2], the question of which model is considered more effective for optimizing skill acquisition remains a recurring topic of debate among sports scientists, sports pedagogues, physical education teachers, and coaches [23, 24].

According to cognitive load theory [25, 26], originally developed for cognitive tasks, which posits that overloading the capacity of working memory hinders performance during the acquisition phase, previous theoretical positions suggested a more beneficial effect during this phase for models allowing repeated task performance (e.g., RL and CI in its blocked form (bCI)) compared to those with rarely repeated successive trials (e.g., CI in its serial (sCI) or random (rCI) forms and DL) [7, 10]. However, this assumption was challenged for the CI model in a recent meta-analysis, pooling more than 100 out-comes from 37 studies, which showed no evidence for better acquisition performance following bCI compared to sCI or rCI [23, 27]. Similarly, prior comparative studies during the acquisition phase provided evidence supporting the superiority of the DL model over the RL model in single movement learning [28–32] and over the CI model in multiple skills learning [14, 22, 29, 32, 33]. These findings also contradict the cognitive overload theory and its related assumptions for non-cognitive tasks.

It is noteworthy that most available comparative studies in this field have focused on coordination-dominated skills, leaving a significant gap in our understanding of the optimal learning model for skills with additional strength aspects. To address this gap, a pioneering study [24] investigated the acute impact of various learning models (e.g. RL, bCI, sCI, and DL) on weightlifting technical efficiency assessed by the barbell pattern and the accompanying neurophysiological response. While the findings offered preliminary support for the benefits of the DL model in terms of acute neurophysiological response to coordination-strength-based exercises in novices, no statistically significant advantage of one learning model over the other was revealed for these specific conditions. The analysis of averaged technical efficiency based on time discrete values showed no significant differences between the learning models [24]. The absence of significant differences between the different practice models in terms of acute technical outcomes, despite the higher cognitive demand following the DL model—evidenced by the more pronounced increase in pulse-related parameters and perceived physical and mental effort—resulting in the lowest changes in brain activation, calls into question a generalization of the cognitive load theory to the non-cognitive field of movements.

While the recent study by Ammar et al. [24] represents a pioneering effort in its domain, it’s important to recognize that its primary focus was on neurophysiological responses after each motor learning bout, with relatively minimal emphasis on biomechanical data analysis. The study analyzed only a few kinematic parameters and did not extend to kinetic parameters, omitting many of the commonly utilized metrics in previous biomechanical studies of weight-lifting. Indeed, Olympic weightlifting movements have frequently been modelled using various time-discrete variables, such as peak average barbell vertical and horizontal displacement, velocity, and acceleration at various instances and in different movement phases [24, 34–38]. This is often combined with kinetic variables analysing force and power-related patterns based on averaged curves of related time-continuous patterns (e.g., peak vertical ground reaction force (vGRF), rate of force development (RFD), power, and work) derived from continuous force-plate data [34, 35, 36]. In training practice, evaluating the broadest possible spectrum of biomechanical variables is often considered optimal for the monitoring of technical efficiency for an individual or a group. Accordingly, the focus on a limited number of biomechanical features in the study of Ammar et al. [24] may restrict a thorough overview of how different motor learning models influence the biomechanics of strength-coordination movements.

Given the scarcity of evidence regarding the acute effect of different motor learning approaches on biomechanical patterns during the acquisition of complex coordination-strength movements, the objective of the present study is to comprehensively evaluate the impact of a learning snatch bout following four distinct motor learning approaches (RL, CI with its blocked (bCI) and serial (sCI) form, and DL) on the most common kinematic and kinetic parameters.

While learning progress is frequently seen after just one bout in learning experiments with few degrees of freedom [10, 27], this is rarely seen in movements with many degrees of freedom, such as in sports contexts. For this reason, interventions lasting several weeks are typically seen in conjunction with repetition learning of sports movements [23]. Nevertheless, the advantages of DL (mostly measured by the performance outcome), which are amplified with increasing intervention duration [39], could already be reflected in shorter interventions with appropriately differentiated measurement of technique performance. Considering the demonstrated advantages of DL practice on brain and heart responses to similar learning bouts [24] and recent findings that suggest the variety of skill variations in DL can reduce frontal lobe overload, thereby aiding motor learning of movements with high degrees of freedom [2, 40], we hypothesize a potential advantage in terms of technical efficiency for the DL model. However, given the acute nature of the current study, we hypothesize that the impact of motor learning models following a single bout of 36 trials on both the kinematic and kinetic patterns of the snatch would be limited.

## MATERIALS AND METHODS

### Participants

The needed sample size was calculated a priori based on procedures suggested by Beck [41] and using the software G∗power [42]. The probability of type I (α ≤ 0.05) and type II (1-β ≥ 0.95) errors was fixed at 0.05, and the assumed correlation between repeated measures was 0.5. Based on the studies of John & Schöllhorn [43] and discussions between the authors, the effect size was set to be 0.5 (medium effect). The analysis revealed that a minimum of 10 participants were considered enough to reduce the likelihood of committing a type 2 statistical error, ensuring an actual power of 95.23%.

Sixteen highly active males (mean age: 23.1 ± 2.1 years, BMI = 24.1 ± 2.2) without experience in the to-be-learned task, were recruited to voluntarily participate in this study. Following the explanation of the protocol, potential risks, and study benefits, participants provided their written consent to engage in this research.

The inclusion criteria for participants are as follows: all participants should be aged between 18 and 29 years old, male, and should have at least 2 years of experience in fitness and/or CrossFit club (i.e., including at least 6 months of performing barbell-based exercises). Participants with prior involvement in Olympic weightlifting, current or past neurological and/or cardiovascular issues, eye disorders, psychiatric conditions, orthopaedic ailments, muscular disorders, and those taking medications that could impact the cardiovascular system were excluded based on the criteria. Furthermore, all participants had no chronic diseases, sleep disturbances, or extreme chronotypes. During the experimental period, participants reported good to very good sleep quality with a very active lifestyle (an average of 966 ± 275 weekly minutes across all physical activities, including walking). The study was conducted according to the Declaration of Helsinki and approved by the local ethics committee of Faculty 02: Social Sciences, Media, and Sport at Johannes Gutenberg University of Mainz. Informed consent was obtained from all participants who were naive to the purpose of the study and were coded with numbers for the anonymity of personal data.

### Experimental design

A randomized within-subject design was conducted to assess the acute effects of single sessions of four different motor learning approaches (i.e., RL, bCI, sCI, and DL). The randomization was performed using a free online software resource (www.randomization.com) (accessed June 10^th^, 2022). Additionally, to ensure balanced groups, participants were matched by their experience levels in fitness and/or CrossFit before randomization. After a familiarization session, participants reported to the laboratory on four separate occasions, with at least a one-week washout period in between [24]. During each test session, a singular motor learning approach was implemented in a randomized order, involving a single training bout. Prior to each training bout, a standardized 10-minute warm-up was performed, including 2 minutes of light jogging, 3 minutes of dynamic stretching (leg swings, arm circles, torso twists), and 5 minutes of activation exercises (bodyweight squats, lunges, push-ups). Each bout comprised 36 trials of power-snatch derivatives, according to one of the four tested motor learning approaches with a 3-minute standardized duration. All testing sessions were conducted in the afternoon, as previously suggested by Ammar et al. [44, 45], to minimize the effect of diurnal biological variations [46]. The measurements were conducted in controlled laboratory conditions, with standardized and minimized changes in brightness, volume, and temperature. Five minutes following each learning session, three power snatch trials with a 20 kg barbell were performed, without instructions, and barbell kinematics and kinetics data were collected. A previous study by Schöllhorn et al. [47] demonstrated that shoe heel heights can influence walking pattern recognition rates, with the highest rates observed using the most extreme heel heights. To control this variable, participants were instructed to wear identical shoes during all test sessions.

### Motor Learning Approaches

The training sessions for the RL method comprised 36 sets of power snatch repetitions. In the case of the two CI approaches (bCI and sCI), the training sessions incorporated not only the power snatch but also two variations: the snatch power jerk (recognized for enhancing overhead strength, stability, balance, and barbell control during the catch-phase), Soriano et al. [48] and the high pull snatch (recognized for improving strength, speed, power, posture, and balance in the snatch extension [49]).

Specifically, the training bout for the CIb approach involved practicing high pull snatch (A), snatch power jerk (B), and power snatch (C) in a blocked order: 12 repetitions of A, followed by 12 repetitions of B, and finally 12 repetitions of C, totalling 36 trials. On the other hand, the training bout for the sCI approach included practising high pull snatch (A), snatch power jerk (B), and power snatch (C) in serial order: ABC repeated 12 times, resulting in a total of 36 trials.

The DL approach incorporated the practice of the three movements in a serial order (ABC × 12, resulting in a total of 36 trials) during the training bout. However, to introduce additional movement variability and enhance fluctuations, various changes were made to different movement and environmental parameters. These adjustments included variations in foot starting position for A, B, and C, barbell starting position for A and C, final positions for B, practising with eyes closed for A, B, and C, and utilizing an unstable surface (using the aeris^®^ muvmat [23]) for A, B, and C. Usually, in DL training, efforts are made to enhance the impact on brain activity and encourage a self-organized learning process by incorporating varied movements and increased fluctuations to make the system unstable [40]. The current approach to power snatch DL identified three levels of movement variability in addition to the reference condition (with no additional movement variability): level 1: variation in only one movement/surrounding parameter; level 2: variation in two movement/surrounding parameters; and level 3: variation in three movement/surrounding parameters.

A nine-second inter-block rest period (12 repetitions) was employed in the RL and both CI conditions, resulting in a total rest period of 18 seconds across 36 trials. Meanwhile, a six-second interset rest period was implemented in the DL condition, totalling 18 seconds across 36 trials. This design aimed to standardize physical (cardiac) exertion in all motor learning approaches, considering their impact on brain activity [50, 51] and the cardiovascular system [52, 53]. This approach allowed us to establish a norm for training session duration, approximately 3 minutes. Moreover, each approach’s training bouts utilized the same empty barbell weighing 10 kg.

### Measurements

#### Kinetic and kinematic measures

The snatch trials were performed on a 2.4 × 0.9 m weightlifting platform and were recorded using Qualisys Track Manager2023.2 (Qualisys AB, Sweden). For the movement kinematics, nine synchronised, commercially available infrared cameras (Oqus 300/310+, Qualisys AB, Sweden) positioned around the platform at a distance of approx. 6 m from the lifting area were used. Two reflective markers were attached to the right and left ends of the barbell, and the average of both markers was calculated to provide movement of the bar center and thus avoid the induction of artefacts associated with asymmetrical movement [34, 35]. The kinematic data were collected at 250 Hz. The calibration was executed beforehand using a calibration wand according to the Qualisys manual.

In addition, the 3D GRF were measured using two Kistler force plates (Type 9287CA; 1000 Hz) (Kistler, Switzerland) embedded in the ground. During the snatch trials, the tested subjects positioned one foot each on a force plate. For this study, the total GRFs were calculated by summing the recorded force vectors of both plates. Before the dynamic snatch trials, a static measurement (without a barbell) was performed to calculate the accurate weight of the subjects.

The recorded data was filtered using a 4^th^-order Butterworth low-pass filter with a cut-off of 4 Hz for the kinematics and 15 Hz for the kinetics. The barbell velocity was calculated by numerical estimation based on the filtered barbell position trajectory. All processed trajectories were normalized to the movement phase from the start of the movement to the catch position [54]. The start position was defined as the time when the vertical barbell velocity was ≥ 0.01 m·s^-1^, and the catch position was defined as the first instance at which the barbel reached a vertical velocity of 0 m · s−1 after the phase of negative vertical velocity following the maximum vertical displacement [24, 54]. The trimmed barbell position and velocity trajectories were normalized based on body height, and the GRF on the basis of body mass. All trajectories were time normalized by linearly interpolating the trajectories to 101 time points (0–100% of the power snatch movement). Finally, the initial values were subtracted from the barbell position trajectories to standardize them, ensuring that all trajectories start from a baseline value of zero.

#### Kinetic and kinematic parameters

Only the anteroposterior (AP) and vertical (V) components were used in the present study, as presented in Figure 1. Barbell kinematic variables were modified from Nago et al. [54], Cunanan et al. [55] and Ammar et al. [24]) and included peak vertical velocity (V-max), peak acceleration (Acc-max), peak barbell height registered during the turnover-phase (Y-max), barbell-height registered during the catchphase (Y-catch), and the vertical travel range (VTR) calculated based on the difference between Y-max and Y-catch (Figure 1). Concerning the horizontal displacement, key parameters included the net horizontal displacement from the start position to the most rearward position during the pull phase toward the lifter (X1), the net horizontal displacement from the start position to the most anterior position during the turnover phase away from the lifter (X2), and the net horizontal displacement from the start position to the catch position (X3) [34, 35] (Figure 1). These horizontal displacements are described as positive-negative-positive or toward-away-toward displacements [35, 46, 56]. Additionally, as presented in Figure 1, the sum of the horizontal displacement during the first and second pulls (DxV) was analyzed along with the horizontal displacement from the most forward position to the catch position (DxL or loop) [34, 56, 57] and the net horizontal displacement from the start position to the catch position (DxT).

**FIG. 1:**
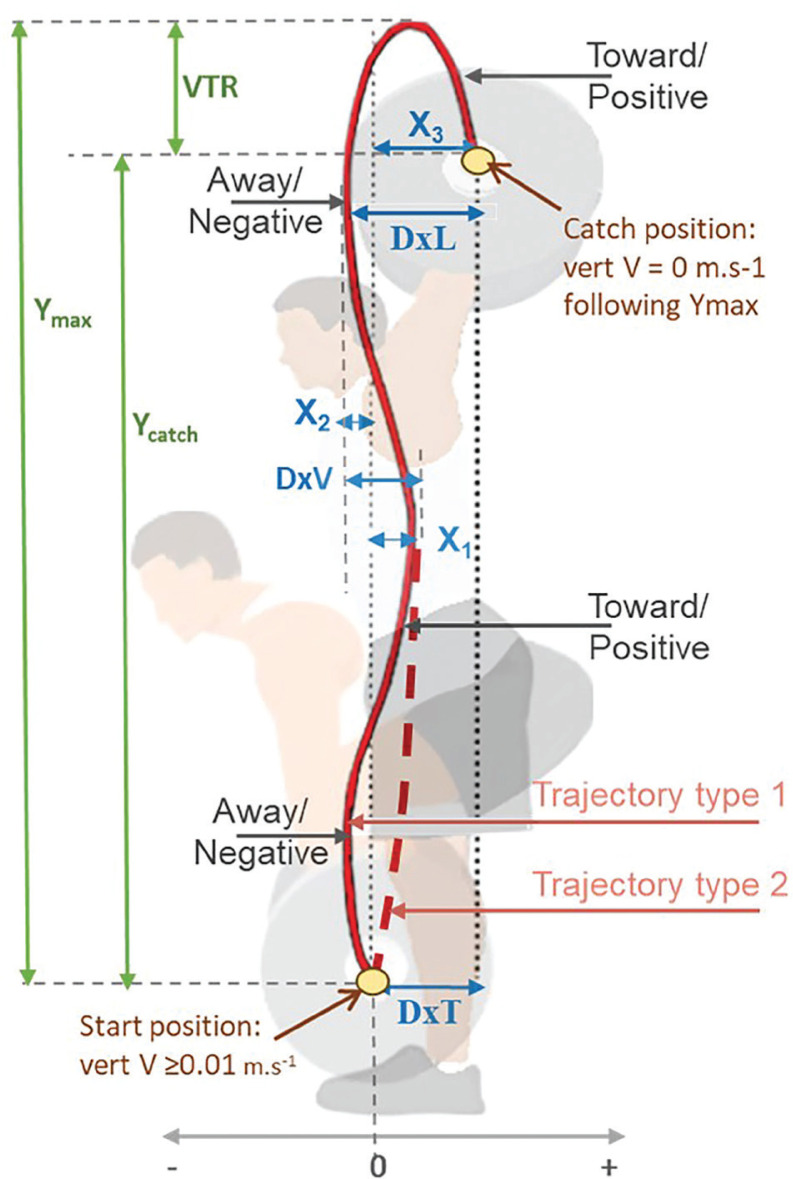
Snatch barbell kinematic variables Peak barbell height (Y-max), barbell-height registered during the catch-phase (Y-catch), and the vertical travel range (VTR), horizontal displacement during the pull phase (X1), horizontal displacement during the turnover phase (X2), horizontal displacement from start position to the catch position (X3/DXT), the sum of the horizontal displacement during the first and second pull (DxV), horizontal displacement from the most forward position to the catch position (DxL).

Regarding the kinetic variables, the analyzed parameters included the peak and average vertical ground reaction force (vGRF) during the whole movement, the peak and average rate of force development (RFD) until the jump, and the peak and average power output during the whole movement. RFD was determined by dividing the difference in consecutive vGRF readings by the time interval (0.001 seconds) between readings and Power was calculated as the product of force and velocity at each time point [35, 46].

#### Statistical analyses

Mean and standard deviation (SD) values were calculated for each variable. The Shapiro-Wilk W-test was used to verify the normal distribution of the data. A one-way repeated measures analysis of variance (RMANOVA) and the Friedman test were used to analyze the main effects of the motor learning model (i.e., 4 levels [motor learning approaches: RL, bCI, sCI, and DL]) on kinematic and kinetic parameters, respectively, for data that were normally and not normally distributed. Post hoc t-tests with Bonferroni correction or Wilcoxon tests were used for pairwise comparison. To estimate the meaningfulness of significant differences, effect sizes were calculated as partial eta-squared ($\eta_{\text{p}}^{2}$) for the main effects. Values of 0.01, 0.06, and 0.13 for partial eta-squared represent small, moderate, and large effect sizes, respectively. Significance was accepted for all analyses at the level of p < 0.05.

## RESULTS

### Effect of motor learning models on Snatch kinematics patterns

The results of the tested kinematics parameters recorded following each learning bout (RL-, bCI-, sCI-, and DL-based bouts) are presented in Table 1. One-way RMANOVA revealed a non-significant effect of group for V-avg, V-max, Acc-max, Y-max, Y-catch, VTR, X1, X2, and DxV with p values ranging from 0.806 to 0.996. Similarly, the Friedmann test showed a non-significant effect of the group for movement duration (p = 0.64), X3/DxT (p = 0.28), and DxL (p = 0.24).

**TABLE 1:** Effect of motor-learning models on snatch kinematics

Variables/motor learning approach	RL	bCI	sCI	DL	ANOVA/Freedman
**MvT duration (s)**	1.29 ± 0.19	1.27 ± 0.27	1.31 ± 0.22	1.32 ± 0.29	test (3) = 1.68 p = 0.642
**V-avg (m · s^−1^)**	1.15 ± 0.25	1.16 ± 0.26	1.13 ± 0.24	1.12 ± 0.29	F (3, 60) = 0.08, p = 0.972
**V-max (m · s^−1^)**	3.18 ± 0.48	3.17 ± 0.35	3.14 ± 0.42	3.15 ± 0.51	F (3, 60) = 0.03, p = 0.995
**Acc-max (m · s^−2^)**	3.18 ± 0.48	3.17 ± 0.35	3.14 ± 0.42	3.15 ± 0.51	F (3, 60) = 0.03, p = 0.996
**Y-max (cm)**	162.06 ± 16.93	158.47 ± 15.88	160.19 ± 18.81	161.44 ± 18.08	F (3, 60) = 0.13, p = 0.941
**Y-catch (cm)**	143.18 ± 21.14	141.11 ± 17.05	143.38 ± 22.24	140.75 ± 19.2	F (3, 60) = 0.08, p = 0.973
**VTR (cm)**	18.88 ± 13.63	17.35 ± 10.43	16.81 ± 10.33	20.69 ± 13.83	F (3, 60) = 0.33, p = 0.806
**X1 (cm)**	-0.88 ± 7.02	-0.21 ± 6.13	-0.43 ± 8.73	0.31 ± 6.74	F (3, 60) = 0.08, p = 0.973
**X2 (cm)**	-16.32 ± 6.3	-16.82 ± 6.97	-15.82 ± 8.04	-16.93 ± 7.33	F (3, 60) = 0.08, p = 0.97
**X3/DxT (cm)**	10.5 ± 8.93	6.93 ± 9.4	9.39 ± 10.21	9.19 ± 9.82	test (3) = 3.83, p = 0.28
**DxL (cm)**	26.81 ± 8.25	23.75 ± 7.17	25.21 ± 8.17	26.12 ± 7.35	test (3) = 4.25, p = 0.236
**DxV (cm)**	15.43 ± 8.51	16.6 ± 9.56	15.39 ± 9.4	17.24 ± 7.6	F (3, 60) = 0.17, p = 0.915

Average vertical velocity (V-avg), Peak vertical velocity (V-max), peak acceleration (Acc-max), peak barbell height (Y-max), barbell-height registered during the catch-phase (Y-catch), and the vertical travel range (VTR), horizontal displacement during the pull phase (X1), horizontal displacement during the turnover phase (X2), horizontal displacement from start position to the catch position (X3/DXT), the sum of the horizontal displacement during the first and second pull (DxV), horizontal displacement from the most forward position to the catch position (DxL).

In terms of barbell trajectory, only DL adhered to the optimal bar-bell path, exhibiting a mean positive value towards the lifter in X1, followed by a negative value away from the lifter in X2, and reverting to a positive value in X3/DXT (Figure 2). For all other models, this optimal trajectory was followed in X2 and X3/DXT, but not in X1, where negative instead of positive mean values were recorded (Figure 2). When examining the barbell paths executed by each subject individually, our analysis indicated that 6 subjects could adhere to the optimal trajectory recommended across all three snatch repetitions following the DL training session. This was followed by the CI models, with 5 and 4 subjects aligning with the optimal path using bCI and serial sCI, respectively, while the RL model shows the lowest adherence, with only 3 subjects following the optimal trajectory.

**FIG. 1:**
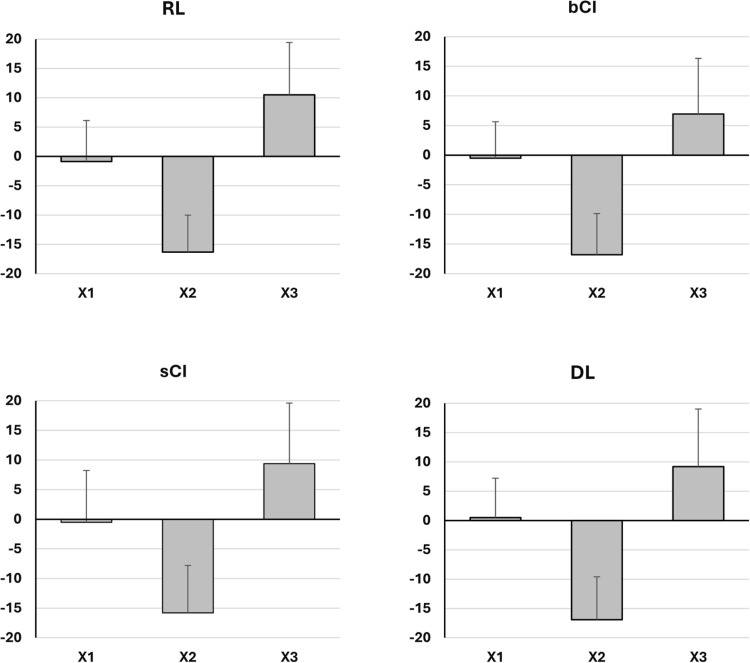
The anteroposterior barbell displacement in responses to the different learning models

### Effect of motor learning models on snatch kinetics patterns

The results of the tested kinetic parameters recorded following each learning bout (RL-, bCI-, sCI-, and DL-based bouts) are presented in Table 2. One-way RMANOVA revealed a non-significant effect of group (p = 0.806 to 0.996) for vGRF mean all, vGRF mean jump, RFD mean all, RFD mean jump, and Power mean jump with p values ranging from 0.887 to 0.95. Additionally, the Friedmann test showed a non-significant effect of group for vGRFmax, RFD max all, RFD max jump, Power max all, and Power mean all with p values ranging from 0.74 to 0.97.

**TABLE 2:** Effect of motor-learning models on snatch kinetics

Variables/motor learning approach	RL	bCI	sCI	DL	ANOVA/Freedman
**vGRF_max-all/jump (N)**	2.59 ± 0.96	2.66 ± 1.3	2.67 ± 1.79	2.55 ± 1.05	test (3) = 0.23, p = 0.973
**vGRF_mean_all (N)**	1.17 ± 0.02	1.17 ± 0.02	1.17 ± 0.03	1.17 ± 0.02	F (3, 60) = 0.1, p = 0.945
**vGRF_mean_jump (N)**	1.4 ± 0.09	1.39 ± 0.09	1.38 ± 0.07	1.39 ± 0.11	F (3, 60) = 0.13, p = 0.944
**RFD_max_all (N · s^−1^)**	149.12 ± 124.05	181.53 ± 189.77	171.47 ± 237.94	165.5 ± 152.79	test (3) = 0.98, p = 0.807
**RFD_max_jump (N · s^−1^)**	10.72 ± 5.93	9.63 ± 4.29	10.96 ± 4.54	10.74 ± 4.58	test (3) = 2.33, p = 0.508
**RFD_mean_all (N · s^−1^)**	-0.02 ± 0.21	-0.07 ± 0.18	-0.07 ± 0.19	-0.05 ± 0.21	F (3, 60) = 0.21, p = 0.887
**RFD_mean_jump (N · s^−1^)**	-2.51 ± 0.64	-2.59 ± 0.76	-2.51 ± 0.79	-2.44 ± 0.69	F (3, 60) = 0.12, p = 0.949
**Power_max_all (W)**	5.14 ± 1.19	5.41 ± 2.19	5.48 ± 3.57	5.34 ± 1.81	test (3) = 0.51, p = 0.917
**Power_max_jump (W)**	4.49 ± 0.8	4.35 ± 0.76	4.38 ± 0.6	4.4 ± 0.81	F (3, 60) = 0.11, p = 0.954
**Power_mean_all (W)**	0.88 ± 0.25	0.88 ± 0.25	0.9 ± 0.26	0.85 ± 0.29	test (3) = 1.27, p = 0.735
**Power_mean_jump (W)**	2.04 ± 0.42	1.97 ± 0.42	1.99 ± 0.51	1.94 ± 0.41	F (3, 60) = 0.16, p = 0.921

Rate of force displacement (RFD) expressed in N/, vertical Ground reaction force (vGRF).

## DISCUSSION

The aim of this study was to analyze the acute effects of single bouts of different motor learning models, namely the RL, bCI, sCI, and DL on snatch technical efficiency and GRF using discrete data from the most common biomechanical monitoring parameters in Olympic weight-lifting. The main findings showed that barbell kinematics and movement kinetics were not statistically significantly affected by the employed learning models during the 36-trial learning bout. No statistically significant differences were observed between these models for any of the 23 parameters tested. These results align with our initial 2^nd^ hypothesis as well as with the findings of Ammar et al. [24], examining snatch barbell trajectory using data from only three related kinematics parameters, and suggesting a limited acute impact of the used variants **FIG. 1.** The anteroposterior barbell displacement in responses to the different learning models of motor learning models on kinematic patterns in novice weightlifters. Considering these variants as representatives of learning approaches may only be plausible for the repetitive and blocked learning methods. Given that rCI and DL models incorporate a variety of concrete realizations, further research is needed to elucidate their specific effects. The theory of “cognitive working memory overload” [26] suggests hindering technical performance during the acquisition phase when employing learning models with high cognitive demand that is typically associated with switching between numerous tasks. DL has been shown to results in higher cognitive demand compared to the other learning models in response to similar learning bout of 36 weightlifting trials, as evidenced by the higher perception of mental demand and the more pronounced decrease in heart rate variability expressed by an increase in low-frequency band [24]. Thus, based on the “cognitive working memory overload” theory, a poorer performance would be anticipated following DL compared to RL, and CI models. However, the lack of significant impact of learning models on snatch biomechanics outcomes revealed by the present findings and in agreement with Apidogo et al. [22] calls this theory into question, at least in the present context. Similarly, recent systematic reviews and meta-analyses, synthesized over 100 acquisition outcomes from 37 CI-related studies within a sports context, further questioned this theory, and demonstrated no evidence of superior acquisition performance for the CI model allowing repeated task performance (bCI) over CI models featuring less frequently repeated successive trials (sCI and rCI) [23, 27].

Notably, the even slightly better barbel trajectory following the present DL compared to RL and CI models, with the DL being the only model to yield an average X1 that aligns with the optimal barbel displacement toward the weightlifter, further challenges this theoretical perspective and suggests a slight non-significant advantage for the DL model.

The observed potential advantages of the DL model on the snatch barbell path in this study are consistent with our initial 1^st^ hypothesis and previous comparative research during the acquisition phase. This body of evidence supports the superiority of the DL model over the RL model in single-movement learning [28–32] and over the CI model in learning multiple skills [14, 22, 29, 32, 33]. These out-comes further challenge the cognitive overload theory and its associated assumptions for non-cognitive tasks. Moreover, they contradict the notion that performing the correct movement as often as possible, as emphasized by the RL and CI models, is necessary for enhanced acquisition performance.

Contrarily, in combination with the advantages on brain activity [24] the present findings in favour of DL propose benefits from learning through the execution of movements with many DGFs, achievable in the DL model through the introduction of added noise [19]. In this study, the snatch learning sessions across all motor learning models were conducted using a 10 kg barbell to mitigate excessive fatigue during the 36 trials, whereas the analyzed snatch trials were performed with a standard Olympic weightlifting barbell weighing 20 kg. Considering the change in barbell weight as a disturbance factor and noting that only under the DL model did the average barbell path (i.e., X1, X2, and X3) follow the recommended trajectory often observed in advanced weightlifters, it appears that for novices, the DL bout provided sufficient noise towards increased resilience to greater disturbances (e.g., change in barbell weight). In contrast, the RL and CI learning bouts failed to introduce sufficient noise, essential for substantial behavioural modification.

Since DL encourages the reinforcement of novel stimuli and noise without repetition, the resulting novelty and unfamiliarity may also enhance the learner’s motivation. The continual confrontation with “novelty” aligns with the theory of “subjective information” from cybernetic pedagogy [2, 58]. This theory posits that while objective information remains largely constant upon exercise repetition, subjective information—which relies on the learner’s prior knowledge—diminishes with each repetition due to increasing redundancy. To maintain or maximize an individual’s learning rate, objective information must continually change to sustain high levels of subjective information for the learner, necessitating the constant presentation of new information. Accordingly, the increased noise in DL appears to resonate with the athlete’s own noise, which can arise from previous experiences (i.e., in the present study achieved during the learning bout) or from the inherent similarities of the practised skills.

It is noteworthy that the noisy training concept in DL shares similarities with the training of artificial neural networks (ANN) within machine learning (ML). Observations in ML have shown that training an ANN with an appropriate level of noise around the learning target enhances robustness in subsequent applications [18, 59]. Therefore, it’s crucial to underline that the optimal level of noise depends on the initial training of the ANNs. In the sports context, determining the optimal amount of noise for achieving the best learning outcomes, especially in learning whole-body movements with a focus on coordination combined with strength, remains an open question requiring further investigation.

In addition to the added noise, it has been previously suggested that the DL advantage over other models might originate from a down-regulation of frontal brain areas to a lower frequency range (i.e., theta and alpha) [40, 60, 61]. In this context, following DL practice, increased theta and decreased beta and gamma frequencies (i.e., higher frequency range) were reported in the frontal lobes of bad-minton learners. In contrast, CI practice was associated with increased gamma and beta frequencies, suggesting increased cognitive stress using the CI model compared to DL [40, 62]. This downregulation following DL practice has been suggested to result from the wide range of skill variations inherent in this model, which may act as a mitigating factor for stress and overload the frontal lobe [40, 60, 61], thus facilitating enhanced motor learning out-comes even in movements with a high DGF [2, 24, 40].

Aiming at validating this explanation across a wider array of sports skills, focusing on coordination-strength, a recent neurophysiological comparative study has sought to determine whether DL practice would lead to a downregulation in frontal brain areas to a lower frequency range through a decrease in high-frequency bands and an increase in low-frequency bands [24]. The main findings were some-what inconclusive, as the weightlifting learning sessions demonstrated the potential to increase alpha, beta, and gamma frequencies across most of the tested brain regions, regardless of the learning model used. However, the studies noted the lowest number of significant increases in beta and gamma frequencies following the DL approach. This was coupled with a significant increase in Heart Rate Variability’s (HRV) normalized low frequency powers and a decrease in HRV’s normalized high frequency powers, offering preliminary evidence of the acute neurophysiological benefits of DL in novices learning coordination-strength movement. Nonetheless, this hypothesis requires further validation across a variety of coordination-strength-based exercises.

The lack of significant impact of motor learning models on the majority of tested snatch kinetic and kinematic parameters may be due to the limited number of learning trials, restricted to a single training bout of 36 trials. Indeed, previous motor learning studies suggested longer practice durations and/or high numbers of total learning-trials to elucidate an impact of practice models on technical efficiency [63, 64], especially in learning complex movement. Although the DL approach has already significantly increased noise compared to RL and bCI, the amount of noise may still have been too small to trigger spontaneous learning. Even larger internal noise could include larger exercise amplitudes in the sagittal plane, asymmetric movement executions, and various initial body positions. External noise could comprise variations of weights (including asymmetric weight distribution), unstable weights such as liquid-filled weights, or combinations of both types of noise. Consequently, further research is required to identify potential training volume thresholds (including total trial numbers and intervention duration) and/or optimal levels of exercise noise to approach the high-performance model of snatch trajectory following the different learning models and to elucidate significant motor-learning model impacts. Additionally, given the very short learning protocol in the present study, consisting of only one learning bout of 36 trials, future research involving longer learning periods, is essential to verify the potential advantages of the DL model on weightlifting technical efficiency [39].

Another reason for the observed limited acute effect of learning bouts on weightlifting technical efficiency in the present study may be the predominance of individual characteristics over motor learning models. This hypothesis draws on the “individuality of whole-body movements” hypothesis, which has been a subject of interest in human movement science for decades [65].

The concept of individuality, incorporating criteria of uniqueness and persistence borrowed from forensic science, has undergone systematic examination and received empirical support through various studies [8, 14, 47, 66–77]. These investigations underscore the challenges in validating such a hypothesis using average oriented and basic inferential statistics on discrete biomechanical parameters due to the persistence issue in learning processes. Consequently, researchers in these studies have proposed adopting more sophisticated approaches to applying the criteria of uniqueness and persistence to the individuality of motor learning processes. Specifically, in the context of motor learning and Olympic weightlifting movements the analysis of continuous data through artificial intelligence techniques, was recommended [23], such as machine learning classification, based on subjects versus motor learning models [78], to substantiate the individuality hypothesis.

### Strengths and Limits

This study is a complementary analysis to the pioneering work by Ammar et al. [24], which assessed the acute effects of various motor learning weightlifting training bouts, with a focus on neurophysiological responses and very limited interest in biomechanical responses. The strength of this study lies in the comprehensive assessment of various kinetic and kinematic parameters (i.e., 23 parameters in total) commonly used to evaluate technical efficiency in Olympic weightlifting.

In general, the limitations of the study are given by the boundary conditions of the study design, and therefore do not allow for generalization. Therefore, it is crucial to interpret the present findings with caution, particularly the time-derived kinematic parameters such as acceleration. Previous reports have suggested that any additional time derivation of kinematic variables (e.g., velocity from displacement or acceleration from velocity) increases the possibility of errors by a factor of ten, especially when these parameters are assessed using video-based kinematic analysis systems [79]. Thus, computing acceleration from time-derived velocity, which is itself derived from the Qualysis displacement data, may yield imprecise results.

Consequently, future studies may benefit from employing other methodologies to confirm these findings. Furthermore, because the present study focused on young adult male novice weightlifters, studies on various populations, such as female or advanced athletes, are needed to understand the learning processes, especially at the beginning of learning.

## CONCLUSIONS

Despite the limited acute impact of the motor learning models on the majorities of kinetic and kinematics snatch parameters, this study offers preliminary evidence supporting the potential acute benefits of the DL model in enhancing technical efficiency among beginners. This is evidenced by the average antero-posterior displacement of the barbell, which matched the commonly recognized optimal displacement. However, to substantiate this slight acute effect, further studies encompassing a broader range of coordination-strength-based exercises are warranted. Additionally, future research involving longer learning periods and a greater number of total learning trials and sessions is essential to verifying the potential mid- and long-term advantages of the DL model in improving technical efficiency in weightlifting.
